# The earliest beetle with mouthparts specialized for feeding on nectar is a parasitoid of mid-Cretaceous Hymenoptera

**DOI:** 10.1186/s12862-021-01930-6

**Published:** 2021-11-22

**Authors:** Jan Batelka, Jakub Prokop

**Affiliations:** Department of Zoology, Faculty of Science, Charles University, Viničná 7, 128 00 Praha 2, Czech Republic

**Keywords:** Burmite, Tenebrionoidea, Ripiphoridae, Nectar feeding mouthparts, Hymenoptera, Aculeata, Parasitism

## Abstract

**Background:**

During the Mesozoic, there were many insects in several holometabolous orders (Neuroptera, Mecoptera and Diptera) with elongated mouthparts adapted for feeding on nectar. The evolutionary history of the megadiverse order Coleptera, which has a great diversity of mouthparts and feeding strategies, is well documented since early Permian with a significant peak in diversity in the Triassic. Currently, however, there is no evidence that in the Mesozoic these beetles fed on nectar despite the recorded specializations for pollination of flowering plants in several families since the mid-Cretaceous.

**Results:**

Here we describe a new wedge-shaped beetle *Melanosiagon serraticornis* gen. et sp. nov. from mid-Cretaceous Burmese amber attributed to Macrosiagonini (Ripiphoridae: Ripiphorinae), which has elongated galea comparable to that in the extant parasitoid genus *Macrosiagon*, and a well known example of adaptation for nectar feeding in Coleoptera. Furthermore, *Salignacicola* gen. nov. is established for *Macrosiagon ebboi* Perrichot, Nel et Néraudeau, 2004, based on the holotype found in mid-Cretaceous amber from France. Systematic positions of both newly established genera are discussed. A list of potential wasp and bee hosts of Ripiphorinae from the Mesozoic is provided.

**Conclusions:**

This study presents evidence of the earliest occurrence of specialized nectar feeding mouthparts in Coleoptera. *Melanosiagon serraticornis* is closely related to extant Macrosiagonini. In all genera belonging to subfamily Ripiphorinae the primary larvae are adapted for parasitism on aculeate Hymenoptera (bees and wasps) and adults are associated with blossoms of flowering plants, in terms of their specialized morphology. Adults of *Macrosiagon* visit blossoms of flowering plants to obtain nectar and lay eggs from which the hatching larvae attack visiting wasps and bees. An association with flowers of some tropical trees is already corroborated in some extant species. Interestingly the larvae of Ripiphorinae are also found in Burmese amber. Thus, both life stages of the mid-Cretaceous Ripiphorinae indicate a close association of this lineage with flowering trees.

**Supplementary Information:**

The online version contains supplementary material available at 10.1186/s12862-021-01930-6.

## Background

Co-radiation of insects and seed plants (spermatophytes) is one of the most intriguing phenomena in evolution with the earliest evidence dating back to the late Paleozoic [1]. Various specializations of insect mouthparts, feeding strategies and interactions particularly with pollination played a crucial role in the evolutionary history of both megadiverse groups [2, 3]. While the Cenozoic is clearly dominated by the radiation of angiosperms, the Mesozoic is primarily characterized by the rise of gymnosperms (conifers, Ginkoaceae and Cycadales) and appearance of angiosperms in the Early Cretaceous, which resulted in new associations between insects and plants as well as host shifts in many insect groups (e.g., [4, 5]. Hence, the Cretaceous is one of the most significant periods in respect of plant–insect co-radiation and evidence of these interactions in both groups is preserved in fossilized resin and as compressed fossils [1, 6]. Among the well-known examples of Cretaceous insect radiations are major anthophilous taxa like the bees (Apidae: Meliponini), pollen wasps (Vespidae: Masarinae), brachyceran flies (Acroceridae, Apioceridae, Bombyliidae, Empididae, Nemestrinidae, Stratiomyidae, and Syrphidae) and moths (Lepidoptera: Micropterygidae, Yponomeutoidea) [1]. Further source of important evidence for the co-evolution is documented as plant–insect interactions in form of feeding traces, mines and galls preserved on leaves (e.g., [7]). While the majority of studies focus on Cretaceous evidence of direct or indirect angiosperm pollinators and/or pollinivores (e.g., [8, 9]) there are only a few on nectivorous insects feeding on floral nectar and pollen of gymnosperms during the Cretaceous (see [10]). The specialized nectar feeding insects with elongated mouthparts first appeared in the Late Paleozoic and diversified remarkably during the Triassic and Jurassic in orders like Mecoptera, Diptera and Neuroptera [10].

Currently, beetles are important pollinators and their Cretaceous record of pollination and pollinivory and co-evolution with gymnosperm and angiosperms is being more and more precisely documented for different lineages (e.g., [4, 11–13]). In contrast, nectar feeding is scarcely documented for extant species of Coleoptera with elongated (proboscis-like) mouthparts, such as Meloidae (*Leptopalpus*, *Nemognatha*, *Gnathium* and *Zonitis*), Ripiphoridae: Ripiphorinae (*Macrosiagon*), Cantharidae (*Chauliognathus*) and less elongated maxillary structures for nectarivory in addition to pollen-feeding as found in Scarabaeidae (Hopliini from Greater Cape Region) (see [14, 15]). Thus, it is not surprising that fossil evidence for such feeding specialization in Mesozoic Coleoptera is so far unknown, although insects with elongated mouthparts adapted for feeding on nectar at that time were much more diverse than in the Cenozoic.

The oldest known representatives of wedge-shaped beetles (Ripiphoridae) are larvae and adults from various mid-Cretaceous deposits of amber in the Northern Hemisphere [16]. Perrichot et al. [17] describe males of two species of Ripiphoridae from the uppermost Albian respective Cenomanian of France as members of Ripiphorinae, however, both these species do not belong to this subfamily. The monotypic genus *Paleoripiphorus* Perrichot, Nel et Néraudeau, 2004 belongs to the Ripidiinae [18, 19], whereas the exact systematic placement of “*Macrosiagon*” *ebboi* Perrichot, Nel et Néraudeau, 2004, within or even outside of the family is debateable [16, 19]. Grimaldi et al. [20] report a syninclusion of five triungulinids as possible Ripiphoridae in Cretaceous Burmese amber. The diagnostic characters of the larvae are the same as those of extant Ripiphorinae and are placed in this subfamily by Batelka et al. [19] with uncertain tribal placement as the only known representatives of this subfamily from the Mesozoic.

Inclusions in Burmese amber in combination with fossils available from other Cretaceous amber deposits is an important source of taxonomic, bionomic, systematic and biogeographic data for Cretaceous Ripiphoridae and other groups of insects (e.g. [21, 22]. Here we report a new genus and species of wedge-shaped beetle from Burmese amber and the first definite adult of a Cretaceous Ripiphorinae with elongated mouthparts adapted for feeding on nectar. We also erect a new genus for mid-Cretaceous “*Macrosiagon*” *ebboi* from France, because this species cannot be attributed to Ripiphorinae as currently defined. Systematic positions of both newly established genera are discussed. In addition, a list of Mesozoic wasps and bees is provided to illustrate the availability of possible hosts for larvae of Cretaceous ripiphorine beetles.

## Methods

### Specimens, locality and geological setting

The holotype of *Melanosiagon serraticornis* gen. et sp. nov. was obtained from amber from the Hukawng Valley, Kachin State, northern Myanmar. Burmese amber, or burmite is dated to the mid-Cretaceous (lower most Cenomanian) age based on U–Pb dating of zircon crystals from the volcaniclastic matrix [23]. The holotype is deposited in the collection of Charles University, Faculty of Science, Department of Zoology, Prague (prefix PřFUK).

The holotype of *Salignacicola ebboi* (Perrichot, Nel et Néraudeau, 2004) is described from amber from Salignac, near Sisteron, Alpes-de-Haute-Provence (France). The dating of this amber is uncertain, probably Cenomanian. The holotype (No. Sis 2.1) is deposited in the Laboratoire de Paléontologie, Muséum National d’Histoire Naturelle, Paris (France).

The plant material was not used in this study.

### Optical devices

Both specimens were studied using an Olympus BX40 microscope, Olympus IX81 inverted fluorescence microscope with UIS2 objectives, Zeiss Lumar V12, Nikon SMZ745T and Leica S9D stereomicroscopes, and photographed using Canon EOS 90D digital camera attached to the microscope or a Leica S9D stereomicroscope. Olympus IX81 was equipped with an ORCA-AG monochromatic 12-bit charge coupled device camera (Hammatsu, Japan). Images taken using the inverse microscope are mirrored. For this, we used the imagej (64-bit) software (Rasband, W.S., ImageJ, U.S. National Institutes of Health, Bethesda, MD, U.S.A.). The original photographs were processed using Adobe Photoshop CS4. Some images were prepared as series of focal layers, which were then combined using the focus-stacking software Helicon Focus (Helicon Soft) or Zerene Stacker (Zerene Systems LLC).

### Taxonomy and terminology

The authorship of the family Ripiphoridae and subfamily Ripiphorinae follows changes proposed by Bouchard and Bousquet [24].

The morphological terminology generally follows Beutel et al. [25] for general body parts, and Kukalová-Peck and Lawrence [26] and Fedorenko [27] for wing venation.

### List of abbreviations

Abbreviations for morphological structures are as follows: ml, median lobe of pronotal disc; ga, galea; md, mandible; pe, posterior edge of pronotal disc; lg, ligula; lbp, labial palpus; mxp, maxillary palpus; and RP, radius posterior.

This publication and the nomenclatural acts it contain are registered in ZooBank, the proposed online registration system for the International Code of Zoological Nomenclature (ICZN). The ZooBank LSIDs (Life Science Identifiers) can be obained and the associated information viewed using any standard web browser by appending LSID to the prefix ‘http://zoobank.org/’. The LSIDs for this publication are: urn:lsid:zoobank.org:pub:04D6E8E8-54D7-46D8-AFD6-6A57AF96A514.

## Results

Order Coleoptera Linnaeus, 1758

Superfamily Tenebrionoidea Latreille, 1802

Family Ripiphoridae Laporte, 1840

Subfamily Ripiphorinae Laporte, 1840

Tribe Macrosiagonini Heyden, 1908.

*Melanosiagon* gen. nov.

Type species. *Melanosiagon serraticornis* sp. nov.

*Etymology*. Generic name is a combination of the black appearance of the specimen (Greek μελάνω) and the generic name *Macrosiagon* [28]. Feminine in gender. The name is registered under ZooBank LSID urn:lsid:zoobank.org:act:46B9D135-5214-4999-9F2B-0BF4133F5F7F.

*Diagnosis.* This genus is characterized by having a long and slender body, long elytra, acute, convex and partially dehiscent, small head, strongly flattened antero-posteriorly with vertex transversely planar, specialized mouthparts with elongated galea, bilobed ligula covered by bristles, prominent tibial spurs, tibial spur formula 2-2-2, antennae 11-segmented, mesonotal and metanotal legs with erect stiff spiniform setae, pretarsal claws serrate, abdominal segments laterally compressed, apical fields on hind wings with secondary “ghost” branches (sensu [26]: 186) and apices of hind wings asymmetrically overlapping in resting position.

*Melanosiagon serraticornis* sp. nov.

*Etymology*. This species epithet refers to the characteristic shape of antennomeres III–X. This species name is registered under ZooBank LSID urn:lsid:zoobank.org:act:9D3499A6-664B-4073-A550-32F96AE5C1B7.

**Fig. 1:**
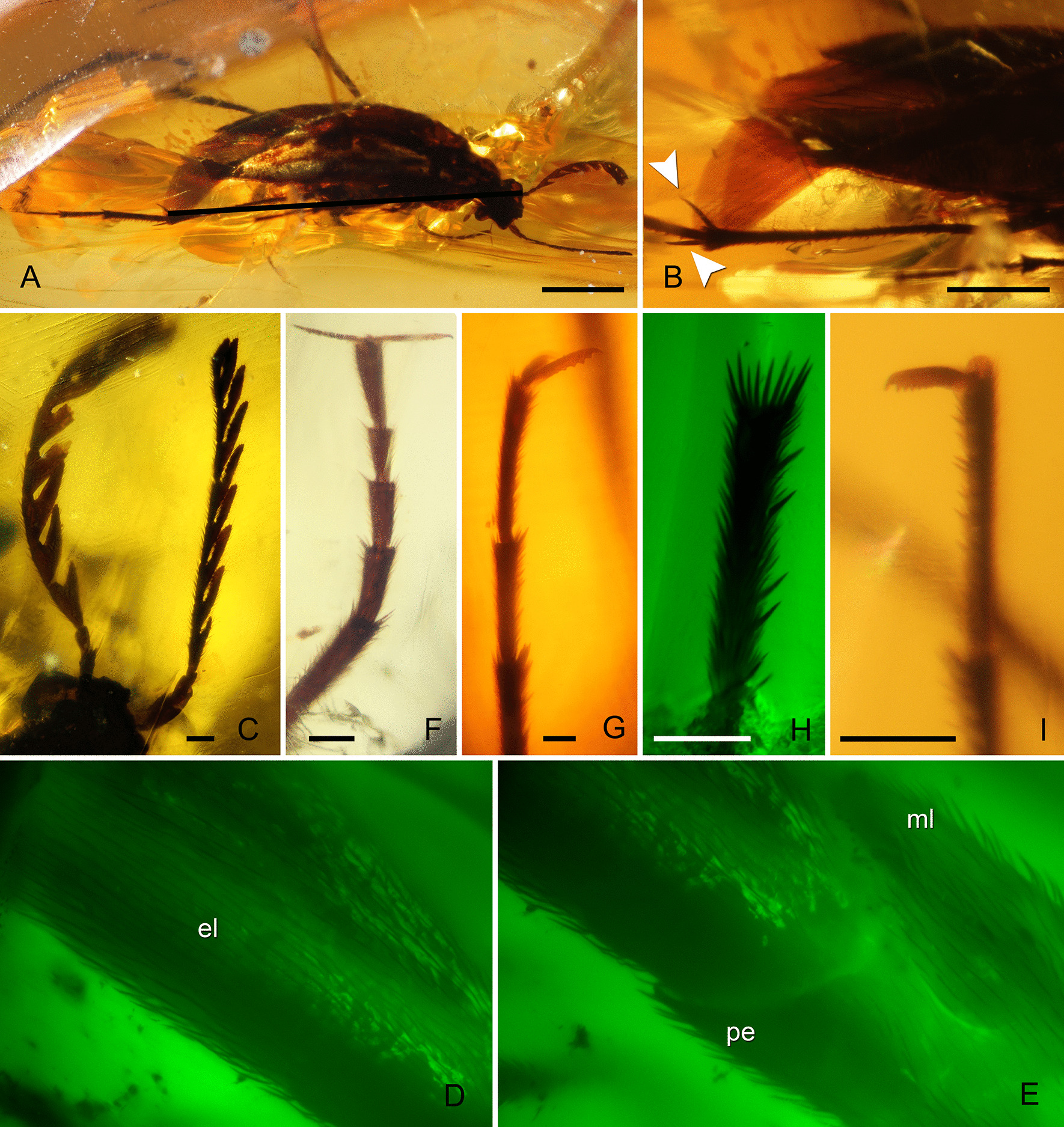
*Melanosiagon serraticornis* gen. et sp. nov. (Ripiphoridae: Ripiphorinae), female, mid-Cretaceous Burmese amber (PřFUK No. 056). **A** Habitus from dorsolateral view. **B** Hindwing apices with secondary “ghost” branches. **C** Antennae with triangular projections on flagellomeres. **D** Detail of elytron viewed under green fluorescence. **E** Detail of pronotal disc viewed under fluorescence. **F** Prothoracic tarsus with five tarsomeres and pretarsal claws. **G** Detail of three distal mesothoracic tarsomeres and serrate pretarsal claws. **H** Mesothoracic tarsomere with erect stiff spiniform setae viewed under green fluorescence. **I** Distal metathoracic tarsomeres and serrate pretarsal claws. *el* elytron, *ml* medium lobe of pronotal disc, *pe* posterior edge of pronotal disc. Scale bars 100 µm (**A**), 50 µm (**B**), 10 µm (**C**, **F**, **G**, **H**, **I**), not in scale (**D**, **E**)

**Fig. 2:**
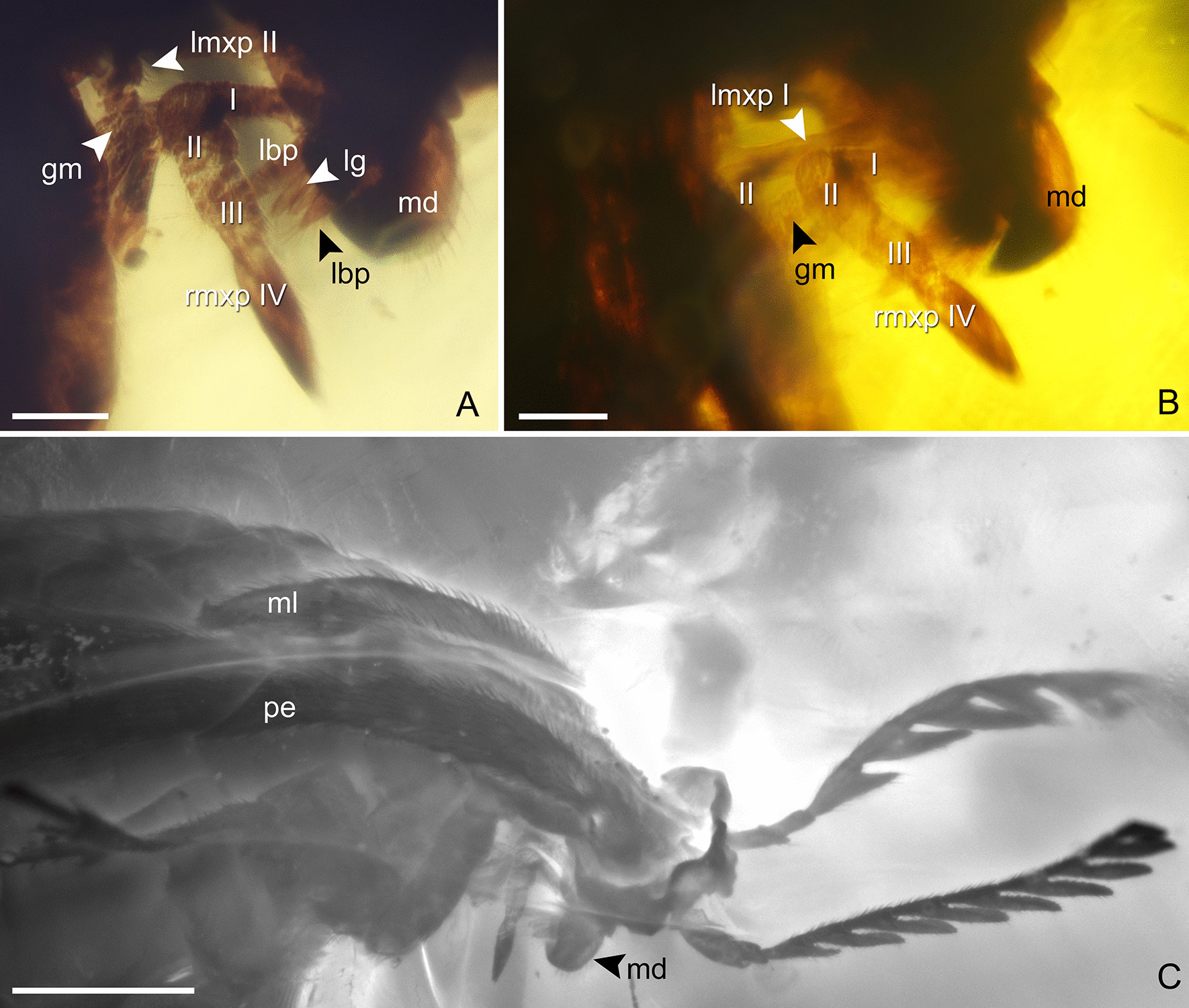
*Melanosiagon serraticornis* gen. et sp. nov., micrographs of head with mouthparts and pronotal disc (PřFUK No. 056). **A**, **B** Mouthparts with elongated galea. **C** Detail of pronotal disc. *gm* galeomere, *lbp* labial palpus, *lg* ligula, *lmxp* left maxillary palpus, *md* mandible, *ml* medium lobe of pronotal disc, *pe* posterior edge of pronotal disc, *rmxp* right maxillary palpus. Scale bars 10 µm (**A**, **B**), 50 µm (**C**)

*Material.* Holotype: PřFUK056 (Figs. 1, 2); lowermost Cenomanian; Myanmar, Kachin, Hukawng Valley; preserved in a polished, transparent yellow piece of amber (14.4 × 10.1 × 0.53 mm). Almost completely preserved female except for four distal pro- and mesotarsomeres and distal half of antennomere XI on the right side (lost during preparation and polishing of the amber). Overall body, especially head capsule and pronotal disc show signs of desiccation of the cuticle and apices of projections on antennomeres VI, VII and XI of the left antenna were broken-off and lost before the body was entrapped in resin.

*Description*. Female. Body dull, black. Head capsule, orthognathous, compressed antero-posteriorly; eyes small, lenticular, prominent in lateral view; vertex transversely planar, slightly elevated above anterior margin of pronotal disc; antennae serrate, inserted in front of eyes; scapus about 2 × as long as pedicel, subcylindrical and slightly curved; pedicel short, about 2 × as long as wide, slightly narrower than scapus; antennomeres III–X distinctly serrate, flattened dorso-ventrally; projections triangular with more or less rounded apices; surface of antennomeres III–XI covered with sparse semi erect sensilla; antennomeres XI similar to preceding ones, albeit not completely preserved; ?labrum dorsally covered by erect setae, mandibles sickle-shaped, ventrally directed, with row of erect setae on their outer lateral edge; labial palpi slightly extending beyond the ligula with terminal palpomere fusiform, apices covered by erect setae; ligula in form of two lobes densely covered by bristles; maxillary palpi 4-segmented; palpomere I not fully discernible, palpomere II longest and widest at apex; palpomere III and IV equal in length; palpomere IV with acute apex; galea distinctly prolonged with surface microstructures probably microtrichia.

Pronotum convex, long, widest at base, distinctly narrower towards apex, covered with sparse semi erect setae; posterior margin of pronotum trilobate; median lobe with shallowly convex ridge reaching from base to 2/3 along length of pronotal disc; elevated process at base of median lobe absent; ventral part of prothorax dark and poorly visible.

Meso- and metathorax wedge-shaped, only poorly visible ventrally; elytra convex from base to tip, dehiscent, blade-shaped, with sharp apices; surface covered with sparse, backward-leaning semi erect setae; outer borders of elytra nearly straight, inner borders of the last quarter curved outwards.

Hind wings dark brown, folded in resting position with asymmetrically overlapping apices, reaching well beyond apices of elytra; apical fields of vein RP with dense longitudinal secondary “ghost” branches.

Legs very long and slender; tarsal formula 5-5-4; pretarsal claws straight, with comb of five short teeth and one longer apical curved tooth; prothoracic legs quite different from meso- and metathoracic legs; claws on pretarsus almost as long as protarsomere V, claws on meso- and metatarsomeres much shorter than respective ultimate tarsomere; each leg with two tibial spurs; tibial spurs short on protibia, long on meso- and metatibia; protarsomeres without distinct apical setal fringe, meso- and metatarsomeres with distinct apical setal fringe except on last tarsomere; protibia and protarsomeres covered with fine setae; meso- and metatibia covered with dense fine setae and sparse longer spiniform setae; meso- and metatarsomeres covered dorsally with fine and dense short setae, but ventrally they are covered with sparse and longer spiniform setae.

Abdomen short with 5 visible segments (sternites III–VII), much shorter than elytra, laterally compressed; abdominal segments tapering posteriorly, each partially the overlapping succeeding one; sternite VII almost acute at apex.

*Measurements*. Total body length as preserved approx. 430 µm; length of antennae approx. 150 µm.

*Remarks*. *Melanosiagon serraticornis* gen. et sp. nov. is easily distinguishable from other Ripiphoridae by the following combination of characters:

1. Head compressed antero-posteriorly with vertex transversely planar and not elevated. This character is present only in some Macrosiagonini: genus *Metoecus* Dejean, 1833 and *Macrosiagon vittata* species group sensu Falin [29].
2. Tibial spur formula 2-2-2. Complete spur formula is preserved only in basal Ptilophorinae (all genera), in *Ivierhipidius* [30] (a highly derived genus of uncertain placement [30], in Ripiphorinae in the genus *Ripiphorus* Bosc, 1791 and *Macrosiagon vittata* species group, and in most New Zealand Pelecotominae (whereas in most of the genera of this subfamily and in all Hemirhipidiinae there is a strong tendency for fewer tibial spurs on all legs [31, 32]).
3. Antennomeres III–X with distinct triangular projections. Serrate or pectinate antennae are common in females of most Ripiphoridae (with some exceptions at the species level). However, in the *Macrosiagon vittata* species group (see above paragraphs 1 and 2) antennal projections are thread-like, as in males of the *Macrosiagon limbata* species group sensu Batelka [33].
4. Elytra convex, dehiscent and covering the whole abdomen. Complete but dehiscent elytra are typical for most of the Macrosiagonini, but they are usually flattened dorsally. Convex elytra are typical for species in the *Macrosiagon limbata* and *vittata* species groups.
5. Apical fields on hind wings with dense longitudinal secondary “ghost” branches. A character occurring only among Ripiphoridae in *Macrosiagon* (e.g., [27]: Fig. A139, [29]: Figs. 1, 2) including the Eocene species *M*. *deuvei* Batelka, Collomb et Nel, 2006 [18]: Figs. 1–3.
6. Hind wings folded in resting position with asymmetrically overlapping apices extending beyond apices of elytra. This character occurs in Ripiphoridae only in *Macrosiagon*. Numerous examples are available for *Macrosiagon*, e.g., [34: Figs. 2, 3], [29: Figs. 1, 2], [18: Figs. 1–3], [33: Figs. 2, 6, 14, 16, 19, 30, 36, 37, 39, 43, 44; [56: Figs. 1, 4, 6, 7] and others. This typical manner of hind wing folding is likely to be due to the narrow width of the wings with reduced venation, ‘compensated’ by secondary “ghost” branches in the apical field, and by a short abdomen and variably dehiscent elytra which usually do not completely cover the hind wings.
7. Meso- and metatibia with spiniform setae. Present also in some Ptilophorinae, but legs in this subfamily are much shorter and stronger, and the apices of tibia are much wider.
8. Long and prominent tibial spurs. Character occurring only in Ptilophorinae and Ripiphorinae, in all other subfamilies there is strong tendency for them to be shorter. In Pelecotominae and Hemirhipidiinae they are even partially hidden in the tibial cavity so they are hardly noticeable under low magnification using a binocular microscope (e.g., [31, 32].
9. Serrate pretarsal claws. Pretarsus with series of distinct teeth are present in Ptilophorinae (their number depends on the size of the specimen (Batelka, pers. obs.)), in *Ripiphorus*, in which the number and size of teeth is a sexual characteristic [35], and in Pelecotominae of South America (formerly Micholaeminae), in which all teeth are very strong and robust (Batelka, unpublished). As the teeth in all the above-mentioned groups differ in shape and number we consider this character as homoplastic, depending possibly on the surface conditions and locomotive requirements of adults. In Cenozoic Macrosiagonini the pretarsal claws are smooth and distinctly bidentate at apex.

Traits of *Melanosiagon* gen. nov. very convincingly place it among Macrosiagonini (see above under characters 1, 4–6); at least characters 4–6 are undoubtedly shared with *Macrosiagon*. Unfortunately, the metathorax in *Melanosiagon* gen. nov. is not discernible so it cannot be compared with Cenozoic Macrosiagonini in which the posterior half of the metepimeron is greatly expanded ventrally above the metacoxa, while the anterior half of the metepimeron evenly tapers towards the mesepimeron. Despite some characters being absent in Cenozoic Macrosiagonini (i.e., serrate pretarsal claws and meso- and metatibia with spiniform setae) we consider *Melanosiagon* gen. nov. to be a member of the Macrosiagonini. Falin [29] doubts the monophyly of *Macrosiagon* in respect to *M*. *vittata* species group and genus *Metoecus*. The phylogenetic analysis of Ripiphoridae based on molecular markers [36] indeed recovered *Metoecus* nested inside *Macrosiagon* (members of *M*. *vittata* species group were not available for this analysis). More data are needed to reveal which species group(s) of *Macrosiagon* should eventually be raised to generic status, equal to *Melanosiagon* gen. nov. and *Metoecus*. If it is accepted that bidentate pretarsal claws are synapomorphy within Cenozoic Macrosiagonini, it seems unlikely that genera in this lineage are descendants of *Melanosiagon* gen. nov.

Family Ripiphoridae Laporte, 1840

Subfamily *incertae sedis.*

*Salignacicola* gen. nov.

Type species. *Macrosiagon ebboi* Perrichot, Nel et Néraudeau, 2004.

*Etymology*. Combination of the name of the type locality and the Latin suffix –*cola* (meaning inhabitant(s)). Masculine in gender. The name is registered under ZooBank LSID urn:lsid:zoobank.org:act:D5E1D0C3-0B1C-4FA6-B2FB-1968308E3B6A.

*Material*. Male holotype preserved as an amber inclusion found in Salignac, near Sisteron (France), Cretaceous (Cenomanian) [17].

*Remarks*. This species is interpreted not to belong to the subfamily Ripiphorinae (to which the genus *Macrosiagon* belongs) by Batelka et al. [19]. After re-examination of the male holotype, this species was tentatively excluded from Ripiphoridae, because of an unusual combination of characters: tibial spur formula 1-2-2; serrate pretarsal claws; putative biflabellate antennae (regarded as uniflabellate by [16: Fig. S3a]) with greatly prolonged thread-like antennal rami covered densely by long hair-like sensilla,reduced venation on hind wings; abdomen long, eight segmented with apex of ninth segment visible (sic), ultimate abdominal segment tapering posteriorly; very long hind legs; and very long metatibial spurs [16: Fig. S3, 17]. Its similarities in terms of characters with other Ripiphoridae considering the description of *Melanosiagon* gen. nov. are updated and discussed below.

Description of *Salignacicola ebboi* (Perrichot, Nel et Néraudeau, 2004), a species originally placed within Macrosiagonini, cannot be compared with other ripiphorids because of absence of some important information. Several of the most prominent characters of this fossil can be, however, preliminarily reconsidered in the context of certain ripiphorid lineages or genera as follows:

1. Tibial spur formula 1-2-2. Reduction in the number of tibial spurs is common in various lineages of Ripiphoridae (see above comments for character 2 under *Melanosiagon* gen. nov.) and occurs also in most Macrosiagonini.
2. Antennomeres III–XI (X) in males with single long projection. Character present in all Ptilophorinae, Pelecotominae and Hemirhipidiinae, and most Ripidiinae (except for several genera with filiform antennomeres and *Paleoripiphorus* with biflabellate antennae). In males of Ripiphorinae antennomeres III–X (IX) always have two long rami.
3. Thread-like antennal rami. Present in some species of *Macrosiagon* (see above comments for character 3 under *Melanosiagon* gen. nov.).
4. Long hair-like sensilla on antennal rami. Similar sensilla occur in several species of *Ptilophorus* Dejean, 1834 inhabiting Socotra, Central Asia and USA [37]. Homology of this character in *Salignacicola* gen. nov. and the modern offshoot *Ptilophorus,* is unlikely.
5. Reduced wing venation. This character state is present in all Ripidiinae [38] and Ripiphorinae, but in both subfamilies the venation is quite different [26, 27].
6. Abdomen is 9-segmented. Ripiphoridae (except for Ripidiinae) have a 5-segmented abdomen. Up to seven visible segments are discernible in some fossil Ripidiinae with VIII^th^ and IX^th^ segment visible as internal structures [38]. In *Salignacicola* gen. nov. the reported number may be incorrect: five translucent telescopically overlapping segments would appear to be nine units. This character should be reinvestigated.
7. Legs long and slender with apical setal fringes on tibia and tarsomeres. Typical for Macrosiagonini (like *Metoecus*), Pelecotominae and Hemirhipidiinae. In Ptilophorinae they are much shorter and stronger, in Ripidiinae distinct tibial and tarsal setal fringes are absent.
8. Long meta(tibial) spurs. Present only in Ptilophorinae and Ripiphorinae (see above comments for character 9 under *Melanosiagon* gen. nov.).
9. Serrate pretarsal claws. Now known in both Ripiphorinae tribes, Ptilophorinae, and South American Pelecotominae (see above comments for character 10 under *Melanosiagon* gen. nov.).

*Salignacicola* gen. nov. shares characters 1, 3, 5, 8 and 9 with Ripiphorinae as the subfamily is now understood (i.e., including *Melanosiagon* gen. nov.), but uniflabellate antennae (character 2) excludes *Salignacicola* gen. nov. from Ripiphorinae more basally or it might require (depending on the shape of the sclerites on the metathorax) redefinition of this character in this subfamily. Long hair-like sensilla on antennal rami (character 4) can be interpreted as an apomorphy of the genus, but not as synapomorphy with certain recent species of *Ptilophorus* bearing similar sensilla. *Salignacicola* gen. nov. thus, may represent a stem lineage of Ripiphorinae (retaining the uniflabellate antennae of its ancestors), which went extinct at the end of Cretaceous, but its exact phylogenetic position within the subfamily will remain unclear until the critical characters of the holotype are reinvestigated using micro-CT and redescribed in accordance with the high standard of the descriptions of most of the Ripiphoridae entrapped in very transparent Burmese amber.

## Discussion

### Coleoptera with maxillae specialized for feeding on nectar

Maxillae (and partially also the labium) in Coleoptera that feed on nectar have attracted the attention of scientists for almost two centuries. The first account of these structures in certain floricolous beetles was presented by Nicholas M. Hentz in 1828 to members of the American Philosophical Society in Philadelphia and published shortly after [28]. He established two new genera, *Chauliognathus* (Cantharidae) and *Macrosiagon* (Ripiphoridae), in which he describes setiferous and penicillate galea, much longer than the maxillary palpus in certain species. However, Hentz also reminds readers of another well-known example of this modification of galea in the meloid genus *Nemognatha* Illiger, 1807. Wilhelmi and Krenn [15] state that proboscis-like mouth parts for feeding on nectar in beetles are found in certain genera of Meloidae, Ripiphoridae and Cantharidae. Another family, which must be added to the list, is Mordellidae, in which several species of anthophilous genera have prolonged galea. The South African genus *Ctenidia* Laporte, 1840 (originally placed in Ripiphoridae) has two segmented, setose and extremely thin galea, longer than half of the length of the head and almost as long as the maxillary palpus [39]. Because males have flabellate and females’ pectinate antennae and both sexes have greatly prolonged galea, a new subfamily, Ctenidiinae was established solely for this genus [40]. Styletous galea also occur in the Palaearctic *Galeimorda* [41], a subgenus of *Variimorda* Méquignon, 1946 established especially because of this peculiar structure [41]. Similar prolonged and acute galea are also described in certain Palaearctic species of *Mordella* Linnaeus, 1758 [42] and *Mordellistena* Costa, 1854 [43]. While the anatomy of maxillae (especially the maxillary palpus) suitable for feeding on nectar was thoroughly investigated in three genera of Meloidae [15], and mouthparts of monotypic *Ctenidia* are described in detail by Franciscolo [39], analyses of the labio-maxillary complex in the speciose genera *Chauliognathus* and *Macrosiagon* are still scattered in the literature and have not been summarized or evaluated. Another specialized pollen and/or nectar feeder are the monkey beetles (Scarabaeidae: Hopliini) characteristic of the floristic hotspot in the Greater Cape Region in South Africa. In this group the mandibles and maxillae are densely bristled and the lacinia mobilis is often toothed and there are bristled galea on the maxillae [14, 44].

### Labio-maxillary complex of Macrosiagonini

The tribe Macrosiagonini contains two extant genera: *Metoecus* and *Macrosiagon*. Yablokov-Khnzoryan [45] figures the maxillae of *Metoecus* with atrophied one-segmented galea, miniaturized lacinia and labium without ligula, which appear to be unsuitable for feeding on plant liquids. This is supported by the experiments and observations of Zahradník [46], that the adults of *M*. *paradoxus* did not consume either pollen grains or nectar, or water with sugar or animal proteins. According to this author, adults only live for 3–4 days and depend exclusively on their body fat.

The situation in *Macrosiagon* is quite different. Mouthparts are specialized for feeding on nectar, although the shapes of the labium and maxilla differ in the different species. Sometimes the representations of these structures in the same species by different authors differ, reflecting either incorrect identification or observation/interpretation errors, or a combination of these errors (Table 1). Hentz [28] established his genus *Macrosiagon* based on the shape of the galea in *M*. *dimidiatum* (Fabricius). The same species was supposedly investigated also by Williams [47] and Falin [48] (but see below). Gerstaecker [49] figures the maxillae of *M*. *spinipennis* (Gerstaecker) and maxillae and labium of *M*. *bimaculatum* (Fabricius). The last species was also studied by Yablokov-Khnzoryan [45]. Manfrini de Brewer [50, 51] figures the maxillae and labium of *M*. *flavipennis* (Leconte) and *M*. *excavatum* (Champion). Maxillae with reduced palpus are figured for *M*. *caffrum* (Fåhraeus) and an unidentified specimen from Zanzibar by Franciscolo [39]. The SEM photomicrographs of mouthparts of an unidentified specimen from USA (which judging from the shape of the head may be *M*. *cruentum* (Germar)) are provided by Großmann [52].

**Table 1:** Review of published descriptions of the labio-maxillary complex of Macrosiagonini

Taxon with reviewed reference	Maxilla	Labium
*Metoecus paradoxus* (Linné), sensu Yablokov-Khnzoryan [45]	One segmented galea atrophied, much shorter than palpomere II; lacinia short, 3 × shorter than palpomere II	Ligula absent
*Macrosiagon spinipennis* (Gerstaecker) sensu Gerstaecker [49]	One segmented galea as long as 4-segmented maxillary palpus; lacinia short, as long as palpomeres I and II combined	Labium not figured
*Macrosiagon bimaculatum* (Fabricius), sensu Gerstaecker [49]	One segmented galea 1.35 × longer than 4-segmented maxillary palpus; lacinia short, as long as palpomeres I and II combined	Ligula as long as 2 distal labial palpomeres, bilobed at apex
*Macrosiagon bimaculatum* (Fabricius), sensu Yablokov-Khnzoryan [45]	Two segmented galea 1.7 × longer than 3-segmented maxillary palpus, lacinia short, shorter than palpomere II	Ligula as long as 2 distal labial palpomeres, bilobed at apex
*Macrosiagon excavatum*(Champion), sensu Manfrini de Brewer [51]	One segmented galea atrophied, much shorter than palpomere II; lacinia prolonged, much longer than palpomeres I-III combined	Ligula almost as long as 2 distal labial palpomeres with two prolonged lobes
*Macrosiagon flavipennis* (Leconte), sensu Manfrini de Brewer [50, 51]	One(?) segmented galea 1.15 × longer than maxillary palpus; lacinia fused with stipes	Ligula absent
*Macrosiagon dimidiatum* (Fabricius), sensu Hentz [28]	One segmented galea 2 × longer than maxillary palpus; distinct lacinia as long as palpomeres I-IV combined	Ligula present (but drawing is too small to be commented in more detail)
*Macrosiagon dimidiatum* (Fabricius), sensu Williams [47]	Two segmented galea 1.4 × longer than maxillary palpus; fused stipes and lacinia shorter than palpomeres I-IV combined	Ligula much shorter than 2 distal labial palpomeres, bilobed at apex
*Macrosiagon dimidiatum* (Fabricius), sensu Falin [48]	Two segmented galea 2 × shorter than maxillary palpus; fused stipes and lacinia shorter than palpomeres I-IV combined	Ligula much shorter than 2 distal labial palpomeres, bilobed at apex
*Macrosiagon caffrum* (Fåhraeus) sensu Franciscolo [39]	One-segmented galea with subclavate apex, lacinia long. Palpus not figured	Labium not figured
*Macrosiagon sp.* (from Zanzibar) sensu Franciscolo [39]	One-segmented galea with clavate apex, lacinia knob-like. Palpus not figured	Labium not figured
*Melanosiagon serraticornis* gen. et sp. nov	Galea at least as long as 2 basal palpomeres	Ligula with two prolonged lobes

In the species studied the labial palpi are always short, three-segmented, ligula very prolonged (but surprisingly absent in *M*. *flavipennis*), always bilobed at apex, with both lobes greatly prolonged (tongue-like) in *M*. *excavatum*. Maxillary palpus of *Macrosiagon* is four-segmented (three-segmented palpus in *M*. *bimaculatum* sensu Yablokov-Khnzoryan [45] is likely an observation error). Lacinia is short, densely covered by setae. Lacinia is not figured as a free (separate) structure in *M*. *dimidiatum* sensu Williams [47] and Falin [48], and is more prolonged than the galea in *M*. *excavatum* sensu Manfrini de Brewer [51] (an observation error?). In the unidentified species from Zanzibar it is figured as atrophied (broken off?) or a knob-like process. Galea is usually acute or narrow at apex, but it is figured clavate in unidentified species from Zanzibar and sub clavate in *M*. *caffrum*.

Number and interpretation of galeomeres vary greatly among authors. Großmann [52] notes that her observation differs from the interpretation of Williams [47]. Großmann [52] shows basal galeomere extremely prolonged and densely covered with setae, and distal galeomere short (only 100 μm long) and smooth with only a few sensilla. She stresses that Williams [47] considers that the basal galeomere is shorter than the distal one, which also ends in a smooth tip. Consequently, Großmann [52] considers Williams [47] observation to be a possible misinterpretation. Falin [48] also interprets the smooth apex of the galea as a “sclerotized tip”, not a separate structure, possibly because the suture between both segments is only visible at a high magnification using SEM. Based on the comparison of all three representatives of *M*. *dimidiatum* summarized in Table 1, there are marked differences in the measurements of particular structures of the maxilla. Despite the possible misinterpretations mentioned above, misidentification of some the specimens should also be considered. Rivnay [53] indicates that *M*. *dimidiatum* is often confused in collections with *M*. *flavipennis* and *M*. *pectinatum* (Fabricius), and sometimes even with *M*. *cruentum*. Thus, future studies on the mouthparts of *Macrosiagon* must pay particular attention to the correct identification of the material examined.

### Ecology and lifestyle of primary larvae and adults of Cretaceous Ripiphorinae

It is likely that the larvae of Ripiphorinae reported by Grimaldi et al. [20] were trapped alive on a tree (because these weakly sclerotized creatures show no signs of desiccation), in contrast, the female of *Melanosiagon serraticornis* gen. et sp. nov. is slightly damaged and desiccated, with its hind wings in a resting position and, therefore, was certainly dead when entrapped in resin (not accidentally flying around a tree and caught by a chance). Both events indicate a close association of the mid-Cretaceous Ripiphorinae with trees producing resin in the area that is nowadays known as northern Myanmar. Albeit many extant species of *Ripiphorus* and *Macrosiagon* lay eggs in blossoms of certain herbaceous plants in semi-desert or desert habitats [33, 54, 55], some species of *Macrosiagon* occur in tropical forests (e.g., [56, 57] and are associated with inflorescences of trees, e.g., *Castanopsis* sp. in Laos [56]. Their role as pollinators should also be considered because they have modified mouthparts with prolonged galea and ligula.

The specialized morphology of triungulinid larvae of Ripiphorinae in Burmese amber indicates a close parasitic relationship with their hosts, possible aculeate Hymenoptera, notably wasps, which are hosts (together with certain lineages of bees) of the extant members of this subfamily. Various groups of wasps (and maybe some bees), which are known as hosts of recent Ripiphorinae, were abundant and diverse around the World during the Cretaceous. Apoidea are recorded in amber, as compressed fossils and there are also nests of Halictidae bees  in Lower and Upper Cretaceous deposits. The following families of Apoidea, Scolioidea, Tiphioidea and Vespoidea are recorded: †Angarosphecidae (15 gen., 50 spp.), †Melittosphecidae (1 gen. et sp.), †Discoscapidae (1 gen. et sp.), Sphecidae (8 gen., 9 spp.), †Cirrosphecidae (1 gen. et sp.), Crabronidae (1 gen. et sp.), Scoliidae (6 gen., 13 spp.), Pompilidae (1 gen. et sp. indet.), Tiphiidae (2 gen., 2 spp.), Vespidae (8 gen., 20 spp.) and Rhopalosomatidae (2 gen., 3 spp.). Except for the last-mentioned family, all the above-mentioned extant families are known to be hosts of extant Ripiphorinae [58]. Thus, Cretaceous wasps and bees may have facilitated the evolution of Ripiphorinae. For detailed list of all the species recorded see Additional file 1.

### Diversity of Cretaceous Ripiphoridae

In addition to the rarely seen Cretaceous Ripiphorinae and *Salignacicola* gen. nov., a genus of uncertain position established herein, the Cretaceous record of other Ripiphoridae is quite rich. In total, five Ripidiinae genera and seven species (including one species of *Paleoripiphorus* indistinguishable from the one found in France) are described from Burmese amber [11, 12, 16, 38, 59]. Unidentified longipede holometabolous primary larvae from Turonian amber of New Jersey, USA reported by Grimaldi et al. [20] are also abundantly recorded in Burmese amber and identified as triungulinids of Ripidiinae [60]. This larval morphotype was later attributed to the genus *Paleoripiphorus* based on the conspecific syninclusion of 28 males, one female (first fossil female of Ripidiinae ever discovered) and two longipede larvae of this genus in Burmese amber [21].

There are also four described genera of Pelecotominae in Burmese amber (for key see [16]), and other morphotypes of triungulinids differing from the longipede morphotype. Grimaldi et al. [20] report a single conicocephalate triungulinid from the Campanian amber of Manitoba (Canada) as a possible strepsipteran larva. Another larva belonging to that conicocephalate morphotype was described subsequently by Beutel et al. [61] from Burmese amber as an early instar of Ripiphoridae without proposing its systematic placement. Kathirithamby et al. [62] reject the conclusions presented in Beutel et al. [61] and his colleagues, and report a third specimen of a conicocephalate larva (again belonging to a different species or even genus) from the Upper Cretaceous amber of Taimyr (Russia) as likely to be a strepsipteran larva. The Cretaceous Strepsiptera in Burmese amber are possibly a monophyletic lineage with four genera [22]. The general morphology of their larvae is identical with that of extant lineages and thus all three conicocephalate morphotypes are considered to be Ripiphoridae [22, 63].

## Conclusions

This study presents evidence of the earliest occurrence of specialized nectar feeding mouthparts in Coleoptera in the mid-Cretaceous ripiphorid *Melanosiagon serraticornis* gen. et sp. nov. in Burmese amber from the Hukawng Valley, Kachin State, northern Myanmar. The mouthparts of which are similar to the 4-segmented maxillary palpi and elongated galea, short labial palpi and bilobed ligula covered by bristles in the extant species of the parasitoid genus *Macrosiagon* [28]. In addition to the structure of the mouthparts *M. serraticornis* has other diagnostic traits of Macrosiagonini, such as the triangular projections on the antennal flagellomeres, long elytra dehiscent posteriorly, tibial spur formula 2-2-2, erect stiff spiniform setae on meso- and metathoracic legs, pretarsal claws serrate, hind wing apices with secondary “ghost” branches and the apices asymmetrically overlap in resting position. Thus, its attribution is well supported. *Salignacicola* gen. nov. is newly established for *Macrosiagon ebboi* Perrichot, Nel et Néraudeau, 2004, based on the holotype found in mid-Cretaceous amber (Cenomanian) from Salignac, Alpes-de-Haute-Provence (France). Inclusion of *Salignacicola* gen. nov. in Ripiphorinae is not supported.

Microstructures like olfactory hair-like sensilla on antennae of *Salignacicola* gen. nov. or microscopic teeth on pretarsal claws and shape of the galea of monotypic *Melanosiagon* gen. nov. are decisive characters for the systematic placement of both new genera. This study demonstrates that fossils must be treated as much as possible in a similar way as their extant relatives if the researcher wants to obtain relevant data about the evolutionary significance and palaeobiology of a particular lineage. Paleoentomology must not rely on superficial similarities of habitus or terminalia, but on a rigorous discussion of the results of investigations of sensory and locomotive microstructures.

The mid-Cretaceous Burmese amber provides exceptionally well-preserved fossils of arthropods and other organisms, which offer excellent insights into the evolutionary history of particular insect groups (for review see, e.g., [22]. The representatives of Ripiphoridae found in Burmese amber are usually very well preserved and can be compared in great detail with their extant relatives. The differences in morphology of these fossil taxa provide a mosaic of important events in the evolution of Ripiphoridae. Based on the extent and increasing quality of the research on Cretaceous specimens over the last two decades, Ripiphoridae could become one of the best represented and studied family of Mesozoic beetles. The present contribution is a small but exciting step towards this objective.

## Supplementary Information

**Additional file 1.** Check-list of Mesozoic wasps and bees.

## Data Availability

All data generated or analysed during this study are included in this published article and its supplementary information file. Specimens investigated are accessible from public institutional collections as specified in “Methods”. New nomenclatural acts were registered in The Official Registry of Zoological Nomenclature open database—ZooBank (http://zoobank.org/).

## References

[1] Grimaldi D (1999). The co-radiations of pollinating insects and angiosperms in the Cretaceous. Ann Missouri Bot Gard.

[2] Labandeira CC (1997). Insect mouthparts: ascertaining the paleobiology of insect feeding strategies. Annu Rev Ecol Syst.

[3] Labandeira CC. The fossil record of insect mouthparts: innovation, functional convergence, and associations with other organisms. In: Krenn HW, editor. Insect mouthparts. Zool. Monographs. 2019; 5, 567–671.

[4] Peris D, Labandeira CC, Barrón E, Delclòs X, Rust J, Wang B (2020). Generalist pollen-feeding beetles during the Mid-Cretaceous. iScience.

[5] Peris D, Pérez-de la Fuente R, Peñalver E, Delclòs X, Barrón E, Labandeira CC (2017). False blister beetles and the expansion of gymnosperm-insect pollination modes before angiosperm dominance. Curr Biol.

[6] Grimaldi DA, Engel MS (2005). Evolution of the insects.

[7] Labandeira CC, Dilcher DL, Davis DR, Wagner DL (1994). Ninety-seven million years of angiosperm insect association: paleobiological insights into the meaning of coevolution. Proc Nat Acad Sci USA.

[8] Grimaldi DA, Peñalver E, Barrón E, Herhold HH, Engel MS (2019). Direct evidence for eudicot pollen-feeding in a Cretaceous stinging wasp (Angiospermae; Hymenoptera, Aculeata) preserved in Burmese amber. Comm Biol..

[9] Huang D, Bechly G, Nel P, Engel MS, Prokop J, Azar D, Cai C-Y, van de Kamp T, Staniczek AH (2016). New fossil insect order Permopsocida elucidates major radiation and evolution of suction feeding in hemimetabolous insects (Hexapoda: Acercaria). Sci Rep.

[10] Khramov AV, Bashkuev AS, Lukashevicha ED (2020). The fossil record of long-proboscid nectarivorous insects. Entomol Rev.

[11] Cai C, Escalona HE, Li L, Yin Z, Huang D, Engel MS (2018). Beetle Pollination of Cycads in the Mesozoic. Curr Biol.

[12] Cai C, Yin Z, Huang D (2018). A new ripiphorid beetle from Upper Cretaceous Burmese amber sheds light on early evolution of the extant subfamily Ripidiinae (Coleoptera: Ripiphoridae). C R Palevol.

[13] Tihelka E, Li L, Fu Y, Su Y, Huang D, Cai C (2021). Angiosperm pollinivory in a Cretaceous beetle. Nat Plants.

[14] Karolyi F, Hansal T, Krenn HW, Colville JF (2016). Comparative morphology of the mouthparts of the megadiverse South African monkey beetles (Scarabaeidae: Hopliini): feeding adaptations and guild structure. PeerJ.

[15] Wilhelmi AP, Krenn HW (2012). Elongated mouthparts of nectar-feeding Meloidae (Coleoptera). Zoomorphology.

[16] Batelka J, Engel MS, Prokop J (2018). A remarkable diversity of parasitoid beetles in Cretaceous amber, with a summary of the Mesozoic record of Tenebrionoidea. Cret Res.

[17] Perrichot V, Nel A, Néraudeau D (2004). Two new wedge-shaped beetles in Albo-Cenomanian ambers of France (Coleoptera: Ripiphoridae: Ripiphorinae). Eur J Entomol.

[18] Batelka J, Collomb FM, Nel A (2006). *Macrosiagon deuvei* n. sp. (Coleoptera: Ripiphoridae) from the French Eocene amber. Ann Soc Entomol Fr.

[19] Batelka J, Prokop J, Engel MS (2016). New ripiphorid beetles in mid-Cretaceous amber from Myanmar (Coleoptera: Ripiphoridae): first Pelecotominae and possible Mesozoic aggregative behaviour in male Ripidiinae. Cret Res.

[20] Grimaldi DA, Kathirithamby J, Schawaroch V (2005). Strepsiptera and triungula in Cretaceous amber. Insect Syst Evol.

[21] Batelka J, Engel MS, Prokop J (2021). The complete life cycle of a Cretaceous beetle parasitoid. Curr Biol.

[22] Pohl H, Wipfler B, Boudinot B, Beutel RG (2020). On the value of Burmese amber for understanding insect evolution: Insights from †*Heterobathmilla—*an exceptional stem group genus of Strepsiptera (Insecta). Cladistics.

[23] Shi G-H, Grimaldi DA, Harlow GE, Wang J, Wang J, Yang M, Lei W, Li Q, Li X (2012). Age constrainton Burmese amber based on U-Pb dating of zircons. Cret Res.

[24] Bouchard P, Bousquet Y (2020). Additions and corrections to “Family-group names in Coleoptera (Insecta)”. ZooKeys.

[25] Beutel RG, Friedrich F, Ge S-Q, Yang X-K. Insect morphology and phylogeny: a textbook for students of entomology. Berlin/Boston: De Gruyter; 2014. p. 516.

[26] Kukalová-Peck J, Lawrence JF (1993). Evolution of the hind wing in Coleoptera. Can Entomol.

[27] Fedorenko DN. Evolution of the beetle hind wing, with special reference to folding (Insecta, Coleoptera). Golovatch SI. editor. Pensoft, Sofia-Moscow;2009. 1–336.

[28] Hentz NM. Remarks on the use of the maxillae in coleopterous insects, with an account of two species of the family Telephoridae, and of three of the family Mordellidae, which ought to be the type of two distinct genera. Trans Am Phil Soc. Philadelphia (NS). 1830; 3, 458–463, pl. XV.

[29] Falin ZH (2004). Revision of three New World *Macrosiagon* Hentz species (Coleoptera: Ripiphoridae: Ripiphorinae) with a discussion of phylogenetic relationships within the Macrosiagonini. Coleopterists Bull.

[30] Barclay MVL (2015). *Ivierhipidius*, an enigmatic new Neotropical genus of Ripiphoridae (Coleoptera: Tenebrionoidea) with four new species. Acta Entomol Mus Nat Pragae.

[31] Batelka J (2009). *Clinopalpus hanae*, a new genus and species of ripiphorid beetle from Malaysia (Coleoptera: Ripiphoridae: Pelecotominae). Acta Entomol Mus Nat Pragae.

[32] Engel MS, Falin ZH, Batelka J (2019). A new genus of Pelecotominae from Mexico, with notes on the genera *Clinops* and *Scotoscopus* and the description of new species (Coleoptera, Ripiphoridae). ZooKeys.

[33] Batelka J (2011). Contribution to the synonymies, distributions, and bionomics of the Old World species of *Macrosiagon* (Coleoptera: Ripiphoridae). Acta Entomol Mus Nat Pragae.

[34] Gressitt JL (1941). Rhipiphoridae from south China (Coleoptera). Ann Entomol Soc Am.

[35] Batelka J (2007). Ripiphoridae (Coleoptera) of Greece and Turkey with notes on their distribution in the Eastern Mediterranean and some neighboring countries. Acta Mus Morav Sci Biol.

[36] Batelka J, Kundrata R, Bocak L (2016). Position and relationships of Ripiphoridae (Coleoptera: Tenebrionoidea) inferred from the ribosomal and mitochondrial molecular markers. Annal Zool.

[37] Batelka J. *Ptilophorus purcharti* sp. nov., the first ripiphorid from Socotra Island, with an account of the biogeography of the Ptilophorini (Coleoptera: Ripiphoridae), pp. 269–285. In: Hájek J, Bezděk J. editors. Insect biodiversity of the Socotra Archipelago. Acta Entomol. Mus. Nat. Pragae. 2012; 52(Suppl 2) i-vi + 1–557.

[38] Batelka J, Prokop J (2019). *Ripidinelia burmiticola* gen. et sp. nov. from Cretaceous amber–the first species of Ripidiinae with tibial spurs (Coleoptera: Ripiphoridae). Paleoentomology.

[39] Franciscolo M (1952). On the systematic position of the genus Ctenidia Castelnau, 1840 (Coleoptera) (Contribution XXXIV [recte: XXIV] to the knowledge of the Mordellidae). Proc R Entomol Soc Lond B.

[40] Franciscolo M. Monografia del genere Pselaphostena mihi (23° Contributo alla conoscenza dei Mordellidae) (Col. Heteromera). Atti Soc. ital. sci. nat., Mus. civ. stor. nat. 1951. Milano 90, 55–76, 1 plate.

[41] Horák J (1985). Ergebnisse der tschechoslowakisch-iranischen entomologischen Expeditionen nach Iran 1970, 1973 und 1977. Coleoptera: Mordellidae 1 (Stenaliini, Mordellini). Entomol Abhandl.

[42] Odnosum VK (2002). Mordellid Beetles (Coleoptera, Mordellidae) in the Fauna of Kazakhstan and Middle Asia. Communication 1. Vest Zool..

[43] Selnekovič D, Goffová K, Kodada J, Improta R (2021). Revealing the identity of Mordellistena minima and *M. pseudorhenana* (Coleoptera: Mordellidae) based on re-examined type material and DNA barcodes, with new distributional records and comments on morphological variability. Can Entomol.

[44] Karolyi F. Chapter 13: What’s on the menu: floral tissue, pollen or nectar? Mouthpart adaptations of Anthophilous Beetles to floral food sources. In: Krenn HW, editor. Insect mouthparts. Zool. Monographs. 2019; 5, 419–442.

[45] Yablokov-Khnzoryan SM (1976). Zhuki-veeronostsy (Coleoptera, Rhipiphoridae) fauny SSSR. II. (Beetles of the family Rhipiphoridae (Coleoptera) of the fauna of the USSR. II) [English translation: Entomol Rev. 1976; 55(2): 104–113.]. Entomol. Obozr..

[46] Zahradník J (1990). *Metoecus paradoxus* (Linné) Coleoptera: Rhipiphoridae—způsob života a hostitelé. [*Metoecus paradoxus* (Linné) Coleoptera: Rhipiphoridae – the way of life and hosts]. Zpr. Západočes. Poboč. Českoslov. Spol. Entomol. Suppl.

[47] Williams IW (1938). The comparative morphology of the mouthparts of the order Coleoptera treated from the standpoint of phylogeny. J N Y Entomol Soc.

[48] Falin ZH. Phylogenetic analysis and revision of the genera and subfamilies of the Ripiphoridae (Coleoptera). Unpublished PhD Dissertation, University of Kansas, Lawrence; 2003. xxiv + 535 pp.

[49] Gerstaecker A. Rhipiphoridum Coleopterorum familiae dispositio systematica. F. Nicolai, Berolini; 1855. 36 pp, 1 pl.

[50] Manfrini de Brewer M (1963). Contribucion al conocimiento de las Ripiphoridae Argentinas (Coleoptera). Opera Lilloana.

[51] Manfrini de Brewer M (1966). Subfamilia Ripiphorinae (Col. Ripiphoridae) revision de las especies Argentinas del genero Macrosiagon Hentz. Rev Prog Cienc Exactas Fis Nat.

[52] Großmann Ch. The head morphology of adult Ripiphoridae (Coleoptera). Unpublished Diploma Thesis, Friedrich Schiller Universität Jena, Biologisch Pharmazeutische Fakultät; 2015. 38 pp.

[53] Rivnay E (1929). Revision of the Ripiphoridae of North and Central America (Coleoptera). Mem Am Entomol Soc.

[54] Batelka J, Straka J (2011). *Ripiphorus caboverdianus* sp. nov.—the first ripiphorid record from the Macaronesian volcanic islands (Coleoptera: Ripiphoridae: Ripiphorinae). Zootaxa.

[55] Linsley EG, MacSwain JW, Smith RF (1952). The life history and development of *Rhipiphorus smithi* with notes on their phylogenetic significance. Univ Calif Publ Entomol.

[56] Batelka J (2013). A review of the genus *Macrosiagon* in Laos (Coleoptera: Ripiphoridae). Ent Basiliensia Coll Frey.

[57] Batelka J, Hoehn P (2007). Report on the host-associations of the genus Macrosiagon (Coleoptera: Ripiphoridae) in Sulawesi (Indonesia). Acta Entomol Mus Nat Pragae.

[58] Batelka J, Chaboo CS (2015). Beetles (Coleoptera) of Peru: a survey of the families. Ripiphoridae Gemminger and Harold, 1870. J Kansas Entomol Soc.

[59] Falin ZH, Engel MS (2010). Notes on Cretaceous Ripidiini and revised diagnoses of the Ripidiinae, Ripidiini, and Eorhipidiini (Coleoptera: Ripiphoridae). Alavesia.

[60] Batelka J, Prokop J, Pohl H, Bai M, Zhang W, Beutel RG (2019). Highly specialized Cretaceous beetle parasitoids (Ripiphoridae) identified with optimized visualization of microstructures. Syst Entomol.

[61] Beutel RG, Zhang WW, Pohl H, Wappler T, Bai M (2016). A miniaturized beetle larva in Cretaceous Burmese amber: reinterpretation of a fossil “strepsipteran triungulin”. Insect Syst Evol.

[62] Kathirithamby J, Perkovsky EE, Falin ZH, Engel MS (2017). A putative twisted-wing parasitoid planidium (Insecta: Strepsiptera) in Taimyr Upper Cretaceous amber. Cret Res.

[63] Pohl H, Batelka J, Prokop J, Müller P, Yavorskaya MI, Beutel RG (2018). A needle in a haystack: Mesozoic origin of parasitism in Strepsiptera revealed by first definite Cretaceous primary larva (Insecta). PeerJ.

