# Supplementary material for: The earliest beetle with mouthparts specialized for feeding on nectar is a parasitoid of mid-Cretaceous Hymenoptera
Source: BMC Ecol Evol. 2021 Nov 22;21:207. doi: 10.1186/s12862-021-01930-6 (PMC8607574; doi:10.1186/s12862-021-01930-6)
Supplement: Supplementary file 1 — Additional file 1. Check-list of Mesozoic wasps and bees. [file 12862_2021_1930_MOESM1_ESM.pdf]

Additional Information

Check-list of Mesozoic wasps and bees (Hymenoptera: Aculeata)

APOIDEA

Halictidae

Genise et al. (2002): nests (Argentina, Central Patagonia, Upper Cretaceous – Santonian, Campanian, compressed fossil)
Genise et al. (2020): nests (Argentina, Patagonia, Lower Cretaceous – Albian, compressed fossil)

†Angarosphecidae

Angarosphex beiboziensis (Hong, 1984) (China, Lower Cretaceous, compressed fossil)
Angarosphex bleachi Rasnitsyn et Jarzembowski, in Rasnitsyn et al., 1998 (UK, Lower Cretaceous, lower Weald Clay, compressed fossil)
Angarosphex consensus Rasnitsyn et Jarzembowski, in Rasnitsyn et al., 1998 (UK, Lower Cretaceous, lower Weald Clay, compressed fossil)
Angarosphex goldringi Jarzembowski, 1991 (UK, Lower Cretaceous, lower Weald Clay, compressed fossil)
Angarosphex lithodes (Zhang, 1985) (China, Lower Cretaceous, Laiyang Fm. Tuanwang, compressed fossil)
Angarosphex lithographicus Rasnitsyn et Ansorge, 2000 (Spain, Lower Cretaceous, La Cabrúa, compressed fossil)
Angarosphex magnus (Darling, 1990) (Brazil, Lower Cretaceous, Santana Formation, compressed fossil)
Angarosphex myrmicopterus Rasnitsyn, 1975 (Russia, Siberia, Baissa, Early Cretaceous, compressed fossil)
Angarosphex niger Rasnitsyn, 1990 (Russia, Turga, Lower Cretaceous, compressed fossil)
Angarosphex pallidus Rasnitsyn, 1986 (Mongolia, Gurvan-Ereniy-Nuru, Lower Cretaceous, compressed fossil)
Angarosphex parvus (Darling, 1990) (Brazil, Lower Cretaceous, Santana Formation, compressed fossil)
Angarosphex penyalveri Rasnitsyn et Martínez-Delclos, 2000 (Spain, Lower Cretaceous, Pedrera de Rúbies, compressed fossil)
Angarosphex saxosus Zhang, Rasnitsyn et Zhang, 2018 (China, Lower Cretaceous, Laiyang Fm., compressed fossil)
Angarosphex strigosus (Zhang, 1992) (China, Lower Cretaceous, Laiyang Fm., compressed fossil)
Angarosphex venulosus (Zhang, 1985) (China, Lower Cretaceous, Laiyang Fm., compressed fossil)
Archisphex boothi Jarzembowski, 1991 (UK, Lower Cretaceous, lower Weald Clay, compressed fossil)
Archisphex catalunicus (Ansorge, 1993) (Spain, Lower Cretaceous, La Cabrúa, compressed fossil)
Archisphex crowsoni Evans, 1969 (UK, Lower Cretaceous, lower Weald Clay, compressed fossil)
Archisphex curvus Rasnitsyn et Jarzembowski, in Rasnitsyn et al., 1998 (UK, Lower Cretaceous, upper Weald Clay, compressed fossil)
Archisphex proximus Rasnitsyn et Jarzembowski, in Rasnitsyn et al., 1998 (UK, Lower Cretaceous, upper Weald Clay, compressed fossil)
?*Baissodes longus* Rasnitsyn, 1990 (Mongolia, Gurvan-Ereniy-Nuru, Lower Cretaceous, compressed fossil)

Baissodes magnus Rasnitsyn, 1975 (Russia, Siberia, Baissa, Early Cretaceous, compressed fossil)
Baissodes robustus Rasnitsyn, 1975 (Russia, Siberia, Baissa, Early Cretaceous, compressed fossil)
?*Baissodes* sp. (Rasnitsyn et al. 1998) (UK, Lower Cretaceous, upper Weald Clay, compressed fossil)
Burmasphex pilosus Melo et Rosa, 2018 (Myanmar, Upper Cretaceous–Cenomanian, amber)
Burmasphex sulcatus Melo et Rosa, 2018 (Myanmar, Upper Cretaceous–Cenomanian, amber)
Cretobestiola communis (Pulawski et Rasnitsyn, in Rasnitsyn et al., 1999) (Russia, Siberia, Baissa, Early Cretaceous, compressed fossil)
Cretobestiola hispanica (Martínez-Delclos et Rasnitsyn, in Rasnitsyn et al., 1999) (Spain, Lower Cretaceous, compressed fossil)
Cretobestiola subpetiolata (Pulawski et Rasnitsyn, in Rasnitsyn et al., 1999) (Mongolia, Bon Tsagan, Lower Cretaceous, compressed fossil)
Cretobestiola tenuipes (Pulawski et Rasnitsyn, in Rasnitsyn et al., 1999) (Russia, Siberia, Baissa, Early Cretaceous, compressed fossil)
Cretosphecium lobatum Pulawski et Rasnitsyn, in Pulawski et al., 2000 (Mongolia, Bon Tsagan, Lower Cretaceous, compressed fossil)
Cretosphecium triste Pulawski et Rasnitsyn, in Pulawski et al., 2000 (Mongolia, Bon Tsagan, Lower Cretaceous, compressed fossil)
Cretosphex incertus Rasnitsyn, 1975 (Russia, Siberia, Baissa, Early Cretaceous, compressed fossil)
?Cretosphex catalunicus Ansorge, 1993 (Spain, Montsec, Lower Cretaceous–Barremian, compressed fossil)
Decasphex cretacicus Zheng, Zhang et Rasnitsyn, 2021 Myanmar, Upper Cretaceous–Cenomanian, amber)
Eubaissodes completus Zhang, 1992 (China, Lower Cretaceous, Laiyang Fm., compressed fossil)
Ilerdosphex wenzae Rasnitsyn, 2000 (Spain, Lower Cretaceous, La Pedrera de Méia, compressed fossil)
Montsecosphex jarzembowskii Rasnitsyn et Martínez-Delclos, 2000 (Spain, Lower Cretaceous, La Pedrera de Rúbies, compressed fossil)
Oryctobaissodes armatus Rasnitsyn, 1975 (Russia, Siberia, Baissa, Early Cretaceous, compressed fossil)
Pompilopterus ciliatus Rasnitsyn, 1975 (Russia, Siberia, Baissa, Early Cretaceous, compressed fossil)
Pompilopterus corpus Rasnitsyn et Jarzembowski, 1998 (UK, Lower Cretaceous, upper Weald Clay, compressed fossil)
?*Pompilopterus difficilis* Rasnitsyn et Jarzembowski, in Rasnitsyn et al., 1998 (UK, Lower Cretaceous, Lulworth Fm., compressed fossil)
Pompilopterus keymerensis Rasnitsyn et Jarzembowski, in Rasnitsyn et al., 1998 (UK, Lower Cretaceous, upper Weald Clay, compressed fossil)
?*Pompilopterus leei* Rasnitsyn et Jarzembowski, in Rasnitsyn et al., 1998 (UK, Lower Cretaceous, upper Weald Clay, compressed fossil)
Pompilopterus montsecensis Rasnitsyn, 2000 (Spain, Lower Cretaceous, La Pedrera de Méia, compressed fossil)
?*Pompilopterus noguerensis* Rasnitsyn et Martínez-Delclos, 2000 (Spain, Lower Cretaceous, La Cabrúa, compressed fossil)
Pompilopterus wimbledoni Rasnitsyn et Jarzembowski, in Rasnitsyn et al., 1998 (UK, Lower Cretaceous, Lulworth Fm., compressed fossil)
?*Pompilopterus worssami* Rasnitsyn et Jarzembowski, in Rasnitsyn et al., 1998 (UK, Lower Cretaceous, upper Weald Clay, compressed fossil)

Trichobaissodes antennatus Rasnitsyn, 1975 (Russia, Siberia, Baissa, Early Cretaceous, compressed fossil)
Vitimosphex incompletus Rasnitsyn, 1975 (Russia, Siberia, Baissa, Early Cretaceous, compressed fossil)
Vitimosphex vividus Zhang, Rasnitsyn et Zhang, 2018 (China, Liutaogou, Lower Cretaceous, compressed fossil)

†Melittosphecidae
Mellitosphex burmensis Poinar et Danforth, 2006 (Myanmar, Upper Cretaceous–Cenomanian, amber)

†Discoscapidae
Discoscapa apicula Poinar, 2020 (Myanmar, Upper Cretaceous–Cenomanian, amber)

Sphecidae
Ampulicinae
Apodolichurus sphaerocephalus Antropov, 2000a (Myanmar, Upper Cretaceous–Cenomanian, amber)
Apodolichurus diaphanus Antropov, 2000a (Myanmar, Upper Cretaceous–Cenomanian, amber)
Cretampulex gracilis Antropov, 2000a (Myanmar, Upper Cretaceous–Cenomanian, amber)
Medampulex monilicularis Antropov, 2000a (Myanmar, Upper Cretaceous–Cenomanian, amber)
Trigampulex pervetus Antropov, 2000a (Myanmar, Upper Cretaceous–Cenomanian, amber)
Ampulicinae gen. et sp. indet. noted by Grimaldi et al. 2002 (Myanmar, Upper Cretaceous–Cenomanian, amber)

†Burmastatinae
Burmastatus triangularis Antropov, 2000a (Myanmar, Upper Cretaceous–Cenomanian, amber**)**

Pemphredoninae
Prolemistus apiformis Antropov, 2000a (Myanmar, Upper Cretaceous–Cenomanian, amber)
Colmepsiterona cumcarena Cockx and McKellar, 2018 (Myanmar, Upper Cretaceous–Cenomanian, amber)
Cretospilomena familiaris Antropov, 2000a (Myanmar, Upper Cretaceous–Cenomanian, amber)
Pemphredoninae gen. et sp. indet. noted by Grimaldi et al. 2002 (Myanmar, Upper Cretaceous–Cenomanian, amber)

Sphecidae subfamily indet.
Frenguelli (1946): nests (Uruguay, Upper Cretaceous–Senonian, compressed fossil)

†Cirrosphecidae
Cirrosphex admirabilis Antropov, 2000a (Myanmar, Upper Cretaceous–Cenomanian, amber)

Crabronidae
Psolimena electra Antropov, 2000b (USA, New Jersey amber, Upper Cretaceous–Turonian)

SCOLIOIDEA
Scoliidae
†Archaeoscoliinae
Archaeoscolia hispanica Rasnitsyn et Martínez-Delclòs, 1999 (Spain, Cuenca, Las Hoyas, Lower Cretaceous– Barremian, compressed fossil)

Proscoliinae

Protoscolia sinensis Zhang, Rasnitsyn et Junfeng, 2002 (China, Beipiao, Liaoning province, Upper Jurassic, Tithonian/lowest Cretaceous, Berriasian, compressed fossil).

Protoscolia imperialis Zhang, Rasnitsyn et Junfeng, 2002 (China, Beipiao, Liaoning province, Upper Jurassic, Tithonian/lowest Cretaceous, Berriasian, compressed fossil)

Protoscolia normalis Zhang, Rasnitsyn et Junfeng, 2002 (China, Beipiao, Liaoning province, Upper Jurassic, Tithonian/lowest Cretaceous, Berriasian, compressed fossil).

Cretaproscolia josai Rasnitsyn et Martínez-Delclòs, 1999 (Brazil, Crato Formation, Lower Cretaceous–Aptian/Albian, compressed fossil)

Cretaproscolia asiatica Zhang, 2006 (China, Laiyang Formation, Shandong, Lower Cretaceous–Barremian, compressed fossil)

Cretoscolia formosa Zhang, 2004 (China, Laiyang Formation, Shandong, Lower Cretaceous–Barremian, compressed fossil)

Cretoscolia laiyangica Zhang, 2004 (China, Laiyang Formation, Shandong, Lower Cretaceous–Barremian, compressed fossil)

Cretoscolia montsecana Rasnitsyn et Martínez-Delclòs, 1999 (Spain, Montsec, Lower Cretaceous–Barremian, compressed fossil)

Cretoscolia promissiva Rasnitsyn, 1993 (Russia, Siberia, Madagan, Upper Cretaceous–Cenomanian, compressed fossil)

Cretoscolia rasnitsyni Zhang, 2004 (China, Laiyang Formation, Shandong, Lower Cretaceous–Barremian, compressed fossil)

Sinoproscolia yangshuwanziensis Zhang, Zhang, Rasnitsyn et Jarzembowski, 2015 (China, Inner Mongolia, Lower Cretaceous, Yixian Formation)

subfamily indet.

Araripescolia magnifica Nel, Escuillie et Garrouste, 2013 (Brazil, Crato Formation, Lower Cretaceous–Aptian/Albian, compressed fossil)

POMPILOIDEA

Pompilidae

Pompilidae gen. et sp. indet. noted by Grimaldi et al. 2002 (Myanmar, Upper Cretaceous–Cenomanian, amber)

TIPHIOIDEA

Tiphiidae

†Thanatotiphiinae

Thanatotiphia nyx Engel, Ortega-Blanco et Bennett, 2009 (Myanmar, Upper Cretaceous–Cenomanian, amber)

?Anthoboscinae

Architiphia rasnitsyni Darling et Sharkay, 1990 (Brazil, Crato Formation, Lower Cretaceous–Aptian/Albian, compressed fossil)

VESPOIDEA

Vespidae

Euparagiinae

Curiosivespa antiqua Carpenter et Rasnitsyn, 1990 (Russia, Siberia, Baissa, Early Cretaceous, compressed fossil)

Curiosivespa curiosa Rasnitsyn, 1975 (Kazakhstan, Kzyl-Zhar, Upper Cretaceous–Turonian)

Curiosivespa derivata Carpenter et Rasnitsyn, 1990 (Mongolia, Bon-Tsagan, Early Cretaceous–?Aptian, compressed fossil)

Curiosivespa striata Perrard et Carpenter, 2017 (Myanmar, Upper Cretaceous–Cenomanian, amber)

Curiosivespa zigrasi Perrard et Carpenter, 2017 (Myanmar, Upper Cretaceous–Cenomanian, amber)

Priorparagia anancites Brothers et Rasnitsyn, 2008 (Botswana, Orapa, Upper Cretaceous–Turonian, compressed fossil)
Celliforma favosites Brown, 1941 ichnosp. (wasp nests) – (USA, Utah, Upper Cretaceous, compressed fossil) (see Brown 1941, Wenzel 1990, Genise 2000)

†Priorvespinae
Alivespa colossa Wu, Shih et Gao, 2020b (Myanmar, Upper Cretaceous–Cenomanian, amber)
Alivespa hirta Wu, Shih et Gao, 2020 (Myanmar, Upper Cretaceous–Cenomanian, amber)
Priorvespa recidiva Carpenter & Rasnitsyn, 1990 (Mongolia, Bon-Tsagan, Early Cretaceous–?Aptian, compressed fossil)
Priorvespa quadrata Carpenter et Rasnitsyn, 1990 (Mongolia, Bon-Tsagan, Early Cretaceous–?Aptian, compressed fossil)
Priorvespa minuta Carpenter et Rasnitsyn, 1990 (Mongolia, Bon-Tsagan, Early Cretaceous–?Aptian, compressed fossil)
Priorvespa directa Carpenter et Rasnitsyn, 1990 (Mongolia, Bon-Tsagan, Early Cretaceous–?Aptian, compressed fossil)
Priorvespa bullata Carpenter et Rasnitsyn, 1990 (Russia, Siberia, Baissa, Early Cretaceous, compressed fossil)
Priorvespa longiceps Carpenter et Rasnitsyn, 1990 (Russia, Central Siberia, Turga, Early Cretaceous, compressed fossil)

†Protovespinae
Protovespa haxairei Perrard et Carpenter, 2017 (Myanmar, Upper Cretaceous–Cenomanian, amber)

Masarinae aff.
Archaeovespa engeli Wu, Shih, Ren et Gao, 2020a (Myanmar, Upper Cretaceous–Cenomanian, amber)
Archaeovespa cretacea Wu, Shih, Ren et Gao, 2020a (Myanmar, Upper Cretaceous–Cenomanian, amber)
Archaeovespa malleata Wu, Shih, Ren et Gao, 2020a (Myanmar, Upper Cretaceous–Cenomanian, amber)

Eumeninae
Symmorphus sennex Carpenter, 2000 (USA, New Jersey, Upper Cretaceous–Turonian, amber)

<u>Rhopalosomatidae</u>
Cretolixon alatum Lohrmann, 2020 (Myanmar, Upper Cretaceous–Cenomanian, amber)
Eorhopalosoma gorgyra Engel, 2008 (Myanmar, Upper Cretaceous–Cenomanian, amber)
Eorhopalosoma lohrmanni Boudinot et Dungey, 2020 (Myanmar, Upper Cretaceous–Cenomanian, amber)
genus and species indet. (fossil fourth instar larva) Lohrmann & Engel, 2017 (Myanmar, Upper Cretaceous–Cenomanian, amber)

Aculeata
Family incertae sedis
Prosphex anthophilos Grimaldi et Engel, 2019 in Grimaldi et al. 2019 (Myanmar, Upper Cretaceous–Cenomanian, amber)

References

Ansorge, J., 1993. Bemerkenswerte Lebenspuren und Cretosphex catalunicus n. sp. (Insecta: Hymenoptera) aus der Unterkretazischen Plattenkalken der Sierra del Montsec (Provinz lerida, NE Spanien). *Neues Jarbuch für Geologie und Paläeontologie, Abhandlungen* **190**: 19–35.

Antropov, A.V., 2000a. Digger wasps (Hymenoptera, Sphecidae) in Burmese amber. *Bulletin of the Natural History Museum, London, Geology* **56**: 59–77.

Antropov, A.V., 2000b. A new digger wasp (Hymenoptera, Sphecidae, Pemphredoninae) from New Jersey amber. pp. 339-343. In: Grimaldi, D.A. (ed.). Studies on fossils in amber, with particular reference to the Cretaceous of New Jersey. Backhuys Publishers Leiden: 1–498.

Antropov, A.V., 2011. A new tribe of fossil digger wasps (Hymenoptera: Crabonidae) from the Upper Cretaceous New Jersey amber and its placement in the subfamily Pemphredoninae. *Russian Entomological Journal* **20**: 229–240. https://doi.org/10.15298/RUSENTJ.20.3.02

Boudinot, B.E., Dungey, D.R., 2020. †*Eorhopalosoma lohrmanni*, a new species of Rhopalosomatidae from mid-Cretaceous amber of northern Myanmar (Hymenoptera, Aculeata: Vespoidea). *Cretaceous Research* **108**: 104339. https://doi.org/10.1016/j.cretres.2019.104339.

Brothers, D.J., Rasnitsyn, A.P., 2008. A new genus and species of Euparagiinae from the Late Cretaceous of southern Africa (Hymenoptera: Vespidae). *Alavesia* **2**: 73–76.

Brown, R.W., 1941. The comb of a wasp nest from the Upper Cretaceous of Utah. *American Journal of Science* (5) **239**: 54–56.

Carpenter, J.M., 2000. A vespid wasp from New Jersey Cretaceous amber. pp. 333–337. *In*: Grimaldi, D.A. (ed.). *Studies on fossils in amber, with particular reference to the Cretaceous of New Jersey*. Backhuys Publishers Leiden: 1–498.

Carpenter, J.M., Rasnitsyn, A.P., 1990. Mesozoic Vespidae. *Psyche* **97**: 1–20.

Cockx, P.F.D., McKellar, R.C., 2018. A new genus and species of the subfamily Pemphredoninae (Hymenoptera: Crabronidae) in Upper Cretaceous amber from Myanmar. *Comptes Rendus Palevol* **17**: 153–157. https://doi.org/10.1016/j.crpv.2017.10.004

Darling, D.C., Sharkey, M.J., 1990. Hymenoptera. pp. 123-153. In: Grimaldi, D.A. (ed.). Insects from the Santana formation, Lower Cretaceous, of Brazil. *Bulletin of the American Museum of Natural History, New York* **195**: 1–191.

Engel, M.S., 2008. The wasp family Rhopalosomatidae in mid-Cretaceous amber from Myanmar (Hymenoptera: Vespoidea), *Journal of Kansas Entomological Society* **81**: 168–174, https://doi.org/10.2317/JKES-712.11.1.

Engel, M.S., Ortega-Blanco, J., Bennett, D.J., 2009. A remarkable tiphiiform wasp in mid-Cretaceous amber from Myanmar (Hymenoptera: Tiphiidae). *Transactions of the Kansas Academy of Science* **112**: 1–6.

Evans, H.E., 1969. Three new Cretaceous aculeate wasps (Hymenoptera). *Psyche* **76**: 251–261. https://doi.org/10.1155/1969/78582.

Frenguelli, J., 1946. Un nido de Esfégido del Cretaceo superior del Uruguay. *Notas del Museo de la Plata* **11**: 259–267.

Genise, J.F., 2000. The ichnofamily Celliformidae for Celliforma and allied ichnogenera. *Ichnos*: *An International Journal of Plant & Animal* **7**: 267–282.

Genise, J.F., Sciutto, J.C., Laza, J.H., Gonzalez, M.G., Bellosi, E.S., 2002. Fossil bee nests, coleopteran pupal chambers and tuffaceous paleosols from the Late Cretaceous Laguna Palacios Formation, Central Patagonia (Argentina). *Palaeogeography, Palaeoclimatology, Palaeoecology* **177**: 215–235. https://doi.org/10.1016/S0031-0182(01)00333-9

Genise, J.F., Bellosi, E.S., Sarzetti, L.C., Krause, J.M., Dinghi, P.A., Sánchez, M.V., Umazano, A.M., Puerta, P., Cantill, L.F., Jicha, B.R., 2020. 100 Ma sweat bee nests: Early and rapid co-diversification of crown bees and flowering plants. *PLoS ONE* **15** (1): e0227789. https://doi.org/10.1371/journal.pone.0227789

Grimaldi, D.A., Engel, M.S., Nascimbene, P.C., 2002. Fossiliferous Cretaceous amber from Myanmar (Burma): its rediscovery, biotic diversity, and paleontological significance. *American Museum Novitates* **3361**: 1–72.

Grimaldi, D.A., Peñalver, E., Barrón, E., Herhold, H.W., Engel, M.S., 2019. Direct evidence for eudicot pollen-feeding in a Cretaceous stinging wasp (Angiospermae; Hymenoptera, Aculeata) preserved in Burmese amber. *Communication Biology* **2**: 408. https://doi.org/10.1038/s42003-019-0652-7

Hong, Y.Ch, 1984. New fossil insects of Laiyang Group from Laiyang Basin, Shandong Province. *Professional Papers of Stratigraphy and Paleontology* **11**: 31–41.

Jarzembowski, E.A., 1991. New insects from the Weald Clay of the Weald. *Proceedings of the Geologists' Association* **102**: 93–108. https://doi.org/10.1016/S0016-7878(08)80069-7.

Lohrmann, V., Zhang, Q., Michalik, P., Blaschke, J., Müller, P., Jeanneau, L., Perrichot, V., 2020. †*Cretolixon* – a remarkable new genus of rhopalosomatid wasps (Hymenoptera: Vespoidea: Rhopalosomatidae) from chemically tested, mid-Cretaceous Burmese (Kachin) amber supports the monophyly of Rhopalosomatinae. *Fossil Record* **23**: 215–236. https://doi.org/10.5194/fr-23-215-2020

Melo, G.A.R., Rosa, B.B., 2018. New genus of fossil apoid wasps (Hymenoptera, Apoidea) from the Cretaceous amber of Myanmar. *Revista Brasileira de Entomologia* **62**: 319–323. https://doi.org/10.1016/j.rbe.2018.09.004

Nel, A., Escuillié, F., Garrouste, R., 2013. A new scoliid wasp in the Early Cretaceous Crato Formation in Brazil (Hymenoptera: Scoliidae). *Zootaxa* **3717**: 395–400. https://doi.org/10.11646/zootaxa.3717.3.10

Perrard, A., Grimaldi, D., Carpenter, J.M., 2017. Early lineages of Vespidae (Hymenoptera) in Cretaceous amber. *Systematic Entomology* **42**: 379–386.

Poinar, G., 2020. Discoscapidae fam. nov. (Hymenoptera: Apoidea), a new family of stem lineage bees with associated beetle triungulins in mid-Cretaceous Burmese amber. *Palaeodiversity* **13**: 1–9. https://doi.org/10.18476/pale.v13.a1

Puławski, W.J., Rasnitsyn, A.P., 2000. *Cretobestiola*, a replacement name for *Bestiola* Pulawski and Rasnitsyn, 1999 (Hymenoptera: Sphecidae). *Journal of Hymenoptera Research* **9**: 209. https://www.raco.cat/index.php/ActaGeologica/article/view/75599.

Rasnitsyn, A.P., 1975. Hymenoptera Apocrita of Mesozoic [Vysshyie pereponchatokrylyie mezozoya]. *Trudy Paleontologicheskogo Instituta. Akademiya Nauk SSSR* (*Transactions of the Palaeontological Institute. Academy of Sciences of the USSR*) **147**: 1–133.

Rasnitsyn, A.P., 1993. Archaeoscolinae, an extinct subfamily of scoliid wasps (Insecta: Vespida = Hymenoptera: Scoliidae). *Journal of Hymenoptera Research* **2**: 85–96.

Rasnitsyn, A.P., 2000. New genus and two new species of the Lower Cretaceous digger wasps from Spain (Hymenoptera: Sphecidae, Angarosphecinae). *Acta Geologica*

Hispanica **35**: 55–58. https://www.raco.cat/index.php/ActaGeologica/article/view/75600.

Rasnitsyn, A.P., Ansorge, J., 2000. New Early Cretaceous hymenopterous insects (Insecta: Hymenoptera) from Sierra del Montsec (Spain). *Paleaontologische Zeitschrift* **74**: 335–341. https://link.springer.com/article/10.1007%2FBF02988105.

Rasnitsyn, A.P., Jarzembowski, E.A., Ross, A.J., 1998. Wasps (Insecta: Vespida Hymenoptera) from the Purbeck and Wealdon (Lower Cretaceous) of southern England and their biostratigraphical and palaeoenvironmental significance. *Cretaceous Research* **19**: 329–391.

Rasnitsyn, A.P., Martínez-Delclòs, X., 1999. New Cretaceous Scoliidae (Vespida = Hymenoptera) from the Lower Cretaceous of Spain and Brazil. *Cretaceous Research* **20**: 767–772.

Rasnitsyn, A.P., Martínez-Delclos, X., 2000. Wasps (Insecta: Vespida = Hymenoptera) from the Early Cretaceous of Spain. *Acta Geologica Hispanica* **35**: 65–95.

Wenzel, J.W., 1990. A social wasp's nest from the Cretaceous period, Utah, U.S.A. and its biogeographic significance. *Psyche* **97**: 21–29.

Wu, Q., Yang, H., Shih, Ch., Ren, D., Zhao, Y. Gao, T., 2020a. Vespids from the mid-Cretaceous with club-shaped antennae provide new evidence about the intrafamiliar relationships of Vespidae. *Zoological Journal of the Linnean Society* **193**: 217–229. https://doi.org/10.1093/zoolinnean/zlaa127

Wu, Q., Zhang, T., Shih, Ch., Ren, D., Zhao, Y. Gao, T., 2020b. New vespids from mid-Cretaceous amber of northern Myanmar provide evidence for the phylogenetic relationships of Priorvespinae (Hymenoptera: Vespidae). *Cretaceous Research* **113**: 104495. https://doi.org/10.1016/j.cretres.2020.104495.

Zhang, J., 1985. New data on the Mesozoic fossil insects from Laiyang in Shandong. *Geology of Shandong* **1**: 23–39.

Zhang, J., 1992. Descriptions of two new genera and two new species of Baissodidae from China (Sphecoidea, Hymenoptera). *Acta Entomologica Sinica* **35**: 483–489.

Zhang, H., Rasnitsyn, A.P., Zhang, J., 2002. The oldest known scoliid wasps (Insecta, Hymenoptera, Scoliidae) from the Jehol biota of western Liaoning, China. *Cretaceous Research* **23**: 77–86.

Zhang, J., 2004. New representatives of Cretoscolia (Insecta: Hymenoptera: Scoliidae) from eastern China. *Cretaceous Research* **25**: 229–234. https://doi.org/10.1016/j.cretres.2003.12.003

Zhang, Q., Rasnitsyn, A.P., Zhang, H.C., 2018a. New Angarosphecidae (Insecta: Hymenoptera: Apoidea) from the Lower Cretaceous of northeastern China. *Paleontological Journal* **52**: 414–420. https://link.springer.com/article/10.1134%2FS0031030118040056

Zheng Y., Chen J., Zhang, H. Rasnitsyn, A.P., 2021. New angarosphecid wasp (Hymenoptera: Apoidea, Angarosphecidae) from the mid-Cretaceous Burmese amber. *Cretaceous Research* **121**: 104742. https://doi.org/10.1016/j.cretres.2020.104742
